# Chinese App User’s Needs Profile: From Questionnaire Measurement to Behavior Analysis

**DOI:** 10.3389/fpsyg.2021.655612

**Published:** 2021-06-18

**Authors:** Zaoyi Sun, Liang Xu, Qi Zhong, Xiuying Qian

**Affiliations:** ^1^College of Education, Zhejiang University of Technology, Hangzhou, China; ^2^Department of Psychology and Behavioral Sciences, Zhejiang University, Hangzhou, China; ^3^College of Mechanical Engineering, Zhejiang University of Technology, Hangzhou, China

**Keywords:** App user, needs, profile, questionnaire, behavior analysis

## Abstract

With the widespread use of mobile devices, the Apps people install and use could be closely linked to their needs. A precise profile of the needs of the user has become a vital foundation of the experience of the user. Previous studies mainly rely on self-reporting to understand the subjective attitudes of the App user toward a single App. This research combined questionnaire measurement and behavior analysis to profile the needs of the App user from a broader perspective. Based on the theoretical model of previous research studies, study 1 developed a novel needs questionnaire measurement of a Chinese App user, which showed good reliability and validity. In study 2, authorized App usage data were collected to construct the behavioral needs profile of a Chinese user. The results showed that the primary needs of the Chinese user remained a relatively high consistency between the questionnaire and the behavior data. The questionnaire-based and behavioral data-based needs profiles provide a reference for further personalized user experience design.

## Introduction

In recent years, the emergence of mobile Internet technology has deeply changed the life style of people. According to a recent report released by the App Annie (2020, Q2) in US, as governments and businesses step up their efforts to stop the coronavirus pandemic, a large number of people started home-based work and learning. As a result, people are spending about 5 h per day on mobile devices on average, an increase of 30% from 2019. The high adoption rate of mobile devices makes Apps an interesting field to study the online communication of people. Based on the uses and gratifications theory (UGT; Katz et al., 1974), the needs of the user were driving forces of social media communication. Recently, some researchers focus on what and how the user chooses Apps to satisfy their needs (e.g., Tanta et al., 2014). For practitioners like App developers, the needs of the user are effective and attractive sources for designing meaningful user experience (Krüger et al., 2017). Thus, how to profile the needs of the user scientifically and effectively has been a topic of widespread concern in academia and industry recently. This study aims to profile the needs of the user from both subjective attitudes and behavior analysis.

Traditional studies usually adopted questionnaires to get quantitative data for the needs of the user. Ji (2013) surveyed Chinese university students about their motivations for using mobile Apps and found that information, social, and entertainment needs were important factors in usage intentions. Kang and Jung (2014) conducted an exploratory factor analysis (EFA) on 398 American and 331 Korean college students. They identified five constructs of the smartphone basic needs scale from the two samples based on Maslow’s hierarchy of needs model (Maslow, 1954). Hsiao et al. (2016) found that hedonic needs (HDNs) were the main factor affecting the intentions of the users to use social mobile Apps. The above studies were based on prior theoretical frameworks and focused on specific Apps. However, the collected questionnaire data were limited in sample size and types of participants (Montjoye et al., 2013).

In contrast to quantitative research studies, qualitative research does not rely on prior theories or models and is more flexible in the choice of subjects and data processing. Grounded theory (GT) is one of the most widely used methods in qualitative research studies, enabling the researchers to seek out and conceptualize the latent social patterns and structures from raw data (Glaser and Strauss, 1967). Some studies have adopted this method to explore the psychological needs structure of a person (e.g., Dukic et al., 2015; Miraglia, 2015). One recent research has specifically used GT to study the needs of the App users (Sun et al., 2017). Coding both the interview data from different age groups and the data from App reviews, the researchers yielded eight types of psychological needs, namely, utilitarian, low-cost, security, health, hedonic, social, cognitive, and self-actualization needs (ANs). However, the experience and biases of researchers may influence the results of GT (Woolley et al., 2000). Although the work of Sun et al. (2017) has considered a wide range of user groups and App categories, more quantitative research data can facilitate better validation of the model.

As mentioned above, qualitative and quantitative research methods not only have their own advantages but also have some limitations in the exploration of psychological phenomena (Blondel et al., 2015). Thus, a mixed method has been advocated. Pincus (2004) summarized the application of mixed methods in the field of consumer psychology that is, conducting qualitative research to elicit consumer marketing issues and identifying consumer language for questionnaire development in quantitative research studies. Recently, some research studies have adopted mixed method to study the use of information technologies (e.g., Walsh, 2014) but have rarely applied it in the field of needs of App user. Therefore, in study 1, we developed a needs questionnaire of App user based on the theoretical model generated by GT (Sun et al., 2017). This questionnaire could not only provide quantitative data for the aforementioned needs model but can also be used for the evaluation of the needs of App user.

For user experience design, questionnaires could help researchers understand the subjective attitudes of the user at the stage of needs assessment (Biduski et al., 2020). At the stage of data analysis, the behavior/objective information of the user would be used for constructing the user profile. Most of the previous research studies collected historical information to infer the usage preference of the user directly (Xin et al., 2015). Li et al. (2014) have pointed that the long-term behavioral preference of the user should be stable. As stable behavioral patterns reflect the unique psychological traits of the user (Shoda et al., 1994), these types of features could solve the “black box” problem of big data to some degree and enhance the interpretability of the model. Recently, a few works have aggregated App behavior records to infer the traits of a user (e.g., Nave et al., 2018), but these works focused on personality. To our knowledge, only one previous work explored the stability of the needs-based profiles of an individual with many anonymous App usage records of the users (Sun et al., 2019). However, there were two limitations of this study. First, they only got access to the cellular data of the user and not wireless fidelity (WiFi) data, which led to a deviation from the actual usage behavior. Second, due to the limitations of big data analysis method, this study presented only aggregated results and did not further compare the results with self-report data of the user.

Therefore, in study 2, we recruited volunteers to participate in a user experience improvement project supported by a Chinese handset company. The participants authorized researchers to anonymously access and record their App usage data of the past month from the back-end data process platform of the company. These data included both WiFi and cellular usage records, having higher ecological validity. Then, the data were analyzed based on the measurement proposed by Sun et al. (2019) to profile the needs of the user. For personality, some studies have found the consistency between behavior prediction and self-report results (Kosinski et al., 2013). Therefore, we tested whether the behavioral needs profile of the user was consistent with the questionnaire data. In doing so, the consistency of behavioral and self-report needs profile put insights into the driven roles of the needs of humans in using the App. Also in practice, this research provided both subjective and objective evaluation of the needs profile of the App user, which could be applied to personalized user experience analysis systems in online communication.

## Study 1: Development of Needs Questionnaire of App User

### Methods

#### Operationalization of the Model

The present study aimed to develop a questionnaire considering needs-related issues of the App user from the aforementioned needs model. The previous research model identified eight types of psychological needs related to App use, namely, utilitarian (e.g., increasing work efficiency and saving time), low-cost (e.g., inexpensive or free), security (e.g., ensuring information security and privacy protection), health (e.g., tracking the physical state and health-related data), hedonic (e.g., fun and leisure experience), social (e.g., facilitating communication and self-expression), cognitive (e.g., information searching and curiosity satisfying), and self-actualization (e.g., improving himself/herself) needs (Sun et al., 2017).

For operationalization of the model, the measurement items were adapted from two aspects. First, after an extensive search for the research studies on the psychological needs, especially in the context of mobile internet, we summarized the relative questionnaires and their constructs. All the measurement items were adapted from prior works of literature. The items of utilitarian needs (UNs) were adapted from the study of Liu et al. (2016). The items of low-cost needs (LCNs) were adapted from the study of Ganesh et al. (2010). The items of security needs (SENs) were adapted from the study of Taormina and Gao (2013). The items of health needs (HENs) were adapted from the study of Zhang et al. (2014). The items of HDNs were adapted from the study of Bondad-Brown et al. (2012). The items of social needs (SNs) were adapted from the study of Haridakis and Hanson (2009). The items of cognitive needs (CNs) were adapted from the study of Wang et al. (2014). The items of self-actualization needs were adapted from the study of Kim et al. (2013). Second, to ensure content validity, we referred to the GT study of Sun et al. (2017) and its detailed online coding appendices. The words of the measurement items of low-cost and self-actualization needs were further adapted from the aforementioned study.

After the above operationalization process, 41 items were measured with a seven-point Likert scale, ranging from 1 (strongly disagree) to 7 (strongly agree). Besides, we adopted the measurement items of the usage attitudes (i.e., “I think smart phone will make life easier in the future”; “I think smart phone will become more and more important in the future”) and intentions (i.e., “I think smart phone will be more frequently used in the future.”) of the smartphone developed by Kang and Jung (2014). The participants were also asked to provide their demographic information, such as age, gender, and occupation.

The above questionnaire was originally created in English, and the validity of the content was discussed by two user experience researchers at the first step. At the second step, two researchers employed back-translation method of Brislin (1970) to ensure translation equivalence. At the third step, 20 university students that had rich App usage experience were invited to rate the clarity and comprehensibility of the items. As a result, the pool of items was reduced from 41 to 35. The final items are presented in the Appendix.

#### Participants and Procedure

The above questionnaire was released on a Chinese online survey platform, and the respondents that finished the survey were rewarded with ¥ 5. In order to test the factor structure of the research model, the original samples were randomly split into two halves (Sample A and Sample B), using the procedure employed by Asendorpf et al. (2001). EFA would be performed on Sample A (*N* = 909) and confirmatory factor analysis (CFA) would be performed on Sample B (*N* = 909).

Table 1 shows the demographic information of the two samples. Compared with the studies recruiting college students, our samples had a wider age and geographical distribution. Specifically, the geographical information covered 29 provinces in China, and Table 1 shows the top five provinces that had the most respondents. In addition, our participants covered different age groups, and almost 70% of them were between 18 and 25. Interestingly, the age distribution was consistent with the age structure of the Chinese Internet users reported by the China Internet Network Information Center (CNNIC, 2018).

**Table 1:** Description of the samples in study 1.

Items	Measure	Sample A	Sample B
		Number of people (Proportion)	Number of people (Proportion)
Gender	Male	341 (37.5%)	403 (44.3%)
	Female	568 (62.5%)	506 (55.7%)
Age	<18 years	45 (5%)	58 (6.4%)
	18–25 years	723 (79.5%)	633 (69.6%)
	26–40 years	114 (12.6%)	172 (18.9%)
	41–50 years	21 (2.3%)	36 (4.0%)
	>50 years	6 (0.7%)	10 (1.1%)
Top-5 locations (Provinces)	Guangdong	425 (46.7%)	323 (35.5%)
	Zhejiang	238 (26.2%)	230 (25.3%)
	HuNan	32 (3.8%)	40 (4.4%)
	JiangSu	29 (3.2%)	38 (4.2%)
	HeNan	28 (3.1%)	40 (4.4%)

### Data Analysis and Results

#### Exploratory Factor Analysis

The questionnaire items in Sample A were preliminary checked for EFA assumptions to be met. The sampling adequacy for performing the analysis was verified through the Kaiser–Meyer–Olkin (KMO) test. The total KMO value was 0.94, which was well above the acceptable limit of 0.60 (Hutcheson and Sofroniou, 1999). Bartlett’s test of sphericity (*χ*2 = 23925.37, *p* < 0.001) indicated that between-item correlations were sufficient to perform EFA. Then, the data of the items were subjected to principal component analyses (PCAs) and a varimax rotation. In the factor analysis, eight factors were extracted and rotated, consistent with the model of Sun et al. (2017). This eight-factor solution was supported by a graphic scree-test and Kaiser’s criterion for number of factors retained (Nunnally and Bernstein, 1994). The eight factors retained accounted for 74.5% of the total variance. Table 2 shows the results.

**Table 2:** Varimax-rotated factor loadings of exploratory factor analysis (EFA; Sample A; *N* = 909).

	SN	HEN	UN	LCN	SEN	HDN	CN	SAN
SN1	**0.76**	0.02	0.19	0.12	−0.05	0.16	0.23	0.11
SN2	**0.81**	0.11	0.07	0.09	0.05	0.17	0.23	0.07
SN3	**0.71**	0.13	0.14	0.10	0.11	0.17	0.16	0.18
HEN1	0.15	**0.76**	0.12	0.06	0.21	0.04	0.09	0.21
HEN2	0.07	**0.89**	0.10	0.06	0.12	0.06	0.08	0.21
HEN3	0.05	**0.89**	0.08	0.06	0.15	0.06	0.05	0.20
HEN4	0.09	**0.82**	0.12	0.10	0.16	0.03	0.09	0.18
HEN5	0.04	**0.70**	0.08	0.10	0.29	0.00	0.03	0.16
UN1	0.11	0.20	**0.81**	0.11	0.17	0.22	0.21	0.11
UN2	0.11	0.19	**0.85**	0.13	0.15	0.27	0.16	0.14
UN3	0.18	0.16	**0.69**	0.17	0.12	0.29	0.22	0.17
UN4	0.16	0.04	**0.70**	0.26	0.07	0.28	0.34	0.16
LCN1	0.16	0.13	0.17	**0.77**	0.02	0.27	0.18	0.13
LCN2	0.12	0.09	0.13	**0.83**	0.10	0.18	0.23	0.15
LCN3	0.05	0.13	0.15	**0.78**	0.15	0.13	0.21	0.07
SEN1	0.04	0.18	0.15	0.15	**0.77**	0.20	0.11	0.19
SEN2	0.05	0.21	0.18	0.16	**0.76**	0.19	0.15	0.22
SEN3	0.04	0.31	0.06	0.03	**0.81**	0.11	0.03	0.24
SEN4	0.03	0.34	0.07	−0.01	**0.77**	0.08	0.04	0.26
HDN1	0.17	0.00	0.13	0.13	0.04	**0.73**	0.23	0.02
HDN2	0.17	0.02	0.16	0.13	0.11	**0.79**	0.22	0.14
HDN3	0.15	0.06	0.22	0.12	0.19	**0.75**	0.23	0.21
HDN4	0.10	0.03	0.13	0.10	0.11	**0.74**	0.28	0.10
HDN5	0.13	0.11	0.21	0.15	0.22	**0.70**	0.17	0.20
HDN6	0.11	0.02	0.23	0.14	0.06	**0.61**	0.41	0.19
CN1	0.15	0.10	0.20	0.20	0.05	0.30	**0.72**	0.16
CN2	0.11	0.08	0.15	0.17	0.13	0.30	**0.71**	0.14
CN3	0.22	0.03	0.16	0.16	0.07	0.26	**0.76**	0.14
CN4	0.16	0.07	0.19	0.15	0.02	0.30	**0.72**	0.16
CN5	0.19	0.13	0.14	0.12	0.12	0.26	**0.67**	0.20
SAN1	0.07	0.20	0.14	0.10	0.15	0.16	0.18	**0.75**
SAN2	0.07	0.21	0.12	0.06	0.19	0.16	0.17	**0.81**
SAN3	0.08	0.25	0.09	0.07	0.20	0.18	0.07	**0.83**
SAN4	0.16	0.19	0.10	0.08	0.16	0.14	0.20	**0.77**
SAN5	0.11	0.25	0.09	0.12	0.24	0.06	0.15	**0.72**

The bold values indicated that each within construct item loading is higher on the measured construct than the cross-loadings on the other items. UN, utilitarian need; LCN, low-cost need; SEN, security need; HEN, health need; HDN, hedonic need; SN, social need; CN, cognitive need; and AN, self-actualization need.

#### Confirmatory Factor Analysis

Confirmatory factor analysis was performed on Sample B (*N* = 909). We evaluated two competitive models: (1) a unidimensional model with all items loading on a general meaning factor and (2) the hypothesized eight-factor model with correlated factors. The values of normed chi (NC)-square, comparative fit index (CFI), goodness-of-fit index (GFI), normed fit index (NFI), and root mean square error of approximation (RMSEA) were compared with the recommended values. The results show that the eight-factor model indicated a good model fit (see Table 3).

**Table 3:** Goodness-of-fit indices from confirmatory factor analysis (Sample B; *N* = 909).

Model	*χ*^2^/*df* (NC)	GFI	CFI	NFI	RMSEA
Unidimensional	15.58	0.54	0.66	0.65	0.13
Eight factors	2.99	0.91	0.96	0.96	0.05
Recommended value	≤3	>0.9			≤0.05

#### Reliability Analysis

Cronbach *α* coefficient was computed for the internal consistency (IC) analysis. Values greater than 0.80 for the Cronbach *α* coefficient of the total questionnaire and greater than 0.70 for the Cronbach α coefficient of each factor were acceptable (Nunnally and Bernstein, 1994). The results in Table 4 show that the estimated reliabilities were acceptable for all eight factors (>0.70). In addition, the Cronbach *α* coefficient of the total questionnaire was 0.95, which was also above the critical point 0.80 level of reliability. In conclusion, the questionnaire had acceptable homogeneity reliability.

**Table 4:** Cronbach *α* coefficient and average variance extracted (AVE) of factors.

	Item numbers	Cronbach *α* coefficient	AVE
Social needs	3	0.80	0.57
Healthy needs	5	0.92	0.72
Utilitarian needs	4	0.86	0.62
Low-cost needs	3	0.87	0.63
Security needs	4	0.91	0.61
Hedonic needs	6	0.91	0.57
Cognitive needs	5	0.90	0.53
Self-actualization needs	5	0.92	0.80

#### Validity Analysis

From the perspective of content validity, the questionnaire items in study 1 were based on the previous questionnaires of needs and were inspired by the language of the interviews of the App user in a work of GT. The understandability and clarity of the contents were also evaluated by the scholars. In addition, the results of EFA and CFA indicated that the questionnaire had good structural validity. Convergent validity measures were assessed by the average variance extracted (AVE). The results in Table 4 show that the AVE of all the factors exceeds the threshold of 0.5 (Fornell and Larcker, 1981).

In terms of criterion-related validity, Kang and Jung (2014) found that the more needs of the user were satisfied through the usage of mobile Apps, the stronger was his/her intention to continue to use mobile phones, and the more positive was his/her attitude toward smartphones. Therefore, the participants in study 1 were also measured about their usage intention and attitudes of the smartphone. Table 5 shows the results of correlation analysis between the needs factors of the mobile App users and their usage intention and attitudes of the smartphone. Each needs dimension was positively correlated with the scores of smartphone use intention and attitude (*p* < 0.001).

**Table 5:** Pearson’s correlation between needs factors and usage intention and attitudes of smartphone.

	Usage intention	Attitude toward smartphones
Healthy needs	0.181^***^	0.218^***^
Security needs	0.304^***^	0.304^***^
Low-cost needs	0.367^***^	0.386^***^
Utilitarian needs	0.473^***^	0.499^***^
Hedonic needs	0.553^***^	0.544^***^
Social needs	0.394^***^	0.440^***^
Cognitive needs	0.523^***^	0.513^***^
Self-actualization needs	0.333^***^	0.404^***^

*** Correlation is significant at the 0.001 level (two-tailed).

## Study 2: Needs Profile of the User Based on Behavior Analysis

In study 2, we attempted to construct the needs profile based on the App usage data and to investigate whether the behavioral needs profile of the individual was consistent with the questionnaire data.

### Participants and Procedure

In this study, we cooperated with a Chinese handset company for a user experience improvement project. We recruited participants that used the mobile phone of this company in online forums. We informed that the participants that, if they took part in the project, they would allow the researchers to access their App usage records of the past month from the back-end data process platform of the company, including records of the App name and timestamp of each usage. The Apps of the users were all downloaded from the Huawei App market and run on Android systems. These data would enter the analysis stages in the form of anonymity, and the whole analysis stages would be completed only on the platform of the company.

The participants that agreed to join in this project needed to complete questionnaire items constructed in study 1 online. At the end of the questionnaire, they were asked to fill in the international mobile equipment identity (IMEI) code of their mobile phone. IMEI is referred as mobile phone “serial number” and is usually used to identify a specific mobile phone in the communication network of an operator (Stutz et al., 2004). All data of the participants were scrutinized and those with the wrong IMEI codes were deleted. Therefore, a valid sample of 30 participants was used for further analysis. Besides, the participant that completed the questionnaire and whose data were successfully collected would be rewarded with ¥50. The above research procedure was reviewed and agreed by the ethics committee of Zhejiang University of Technology. Thirty-two participants who agreed to participate in the study signed an electronic informed consent.

### Calculation

#### Questionnaire Data

The psychological needs questionnaire of the mobile App users in study 1 contained eight measurement items of psychological needs dimensions. Items in each dimension were averaged, with higher scores representing higher levels of the construct.

#### App Usage Data

First, each participant *n*’s 30-day App usage data were collected from the back-end data process platform of the company, including the name of the App, timestamps, and the number of usage times. Based on the previous research methods (Sun et al., 2017, 2019), the probability distribution of each App k on eight needs dimensions was also obtained as:

$$E^{K}={\left[e_{0}^{k},e_{1}^{k},\ldots ,e_{7}^{k}\right]}^{T}$$

where $0\leq e_{i}^{k}\leq 1,\overset{i=0}{\underset{7}{\sum }}e_{i}^{k}=1$. The data format after convergence was as follows:

User_id|App id|Needs tag|use_times ($\left.n\right|k\left|E^{k}\left|C_{nk}\right.\right.$).

Then, we calculated the App *k*’s average use times $\;\mu_{k}$ and the SD $\;\sigma_{k}$. After that, for each user *n*, his/her actual use times, *m*, was combined to calculate the *Z* score *Z_nk_* of each used App as:

$$Z_{nk}=\frac{m-\mu_{k}}{\sigma_{k}},\left(n=1,\ldots 30\right),\left(k=1,2,\ldots y\right)$$

where *y* is the number of Apps used by user *k*.

Finally, based on the method of Zhang and Yu (2015), weighted by *Z_nk_*, we calculated user *n*’s needs dimension *i′* s weighted mean score vector *P_ni_* as:

$$P_{ni}=\frac{\sum_{k=1}^{y}\left(Z_{nk}\right.\cdot e_{ik})}{\sum_{k=1}^{y}Z_{nk}},\left(n=1,\ldots 30\right),\left(i=0,1,\ldots 7\right),\left(k=1,2,\ldots y\right)$$

### Results

#### Descriptive Statistics

Table 6 shows the demographic characteristics of the participants. About 86.7% participants were 18–65 years old (age: *M* = 22.39, *SD* = 3.08), and 83.4% had more than 3 years experience of using mobile phone. These young and experienced users could bring more information for our research. Besides, the Apps used by these participants contained a wide range of categories, such as social media, games, online-shopping, and so on. Besides, the number of App categories used by the individual ranged from 7 to 13, which contained most categories in Huawei App markets, including social media Apps, game Apps, online shopping Apps, and so on. Therefore, our following findings could be generalized.

**Table 6:** Demographic information of participants in sample 2 (*N* = 30).

Measure	Items	Number of people (Proportion)
Gender	Male	9 (30%)
	Female	21(70%)
Age	18–25 years	26(86.7%)
	26–40 years	1(3.3%)
	41–50 years	1(3.3%)
	>50 years	2(6.7%)
Experience of using mobile phone	<12 months	1(3.3%)
	1–3 years	4(13.3%)
	>3 years	25(83.4%)

#### Results of Primary Needs of the Individual

As questionnaire and App usage needs scores of the individual had different ranges and calculation rules, instead of a direct comparison of the values, we ranked each type of scores from high to low separately and calculated the primary needs of each participant (the needs ranked first). First, for the questionnaire scores, since more than one needs had the same value, there were in total 34 primary needs among all the 30 participants. As shown in Table 5, for all the primary needs of the participants, the top three were utilitarian, low-cost, and HDNs, and the proportion of health and SENs was relatively low. Besides, no participant rated the cognitive needs as their primary needs. Although the self-actualization need was at the highest level in Maslow’s hierarchy of needs theory, in this research, this need was rated four times (8%) as the primary need of all participants.

Then, we ranked the scores calculated through the App usage data of the participant. Since, in this case, the eight rankings of each participant were all unique, there were in total 30 primary needs among all the participants. As shown in Table 7, for all the primary needs of the participants, the top two were utilitarian and HDNs. This result was consistent with Nielsen’s report about the primary motivations of the App user (Nielsen and Budiu, 2013). Compared with the results of questionnaire data, we found that although cognitive needs were not regarded as the primary needs in subjective evaluation of any user, it was calculated as the primary needs of three participants (10%) from the App usage data. In contrast, the self-actualization needs were not the primary needs of any participant from the App usage data, although, in subjective evaluation, it was rated as the primary need four times.

**Table 7:** Descriptive statistics for primary needs of the participants (the needs ranked first).

Type of needs	Questionnaire scores		Usage data scores	
	Frequency	Percentage (%)	Frequency	Percentage (%)
Utilitarian	9	26.4%	15	50%
Low-cost	8	23.6%	2	6.7%
Hedonic	7	20.6%	4	13.3%
Social	5	14.7%	3	10%
Self-actualization	3	8.9%	0	0
Cognitive	0	0	3	10%
Security	1	2.9%	0	0
Health	1	2.9%	3	10%

#### Needs Profile Analysis

**Figure 1:**
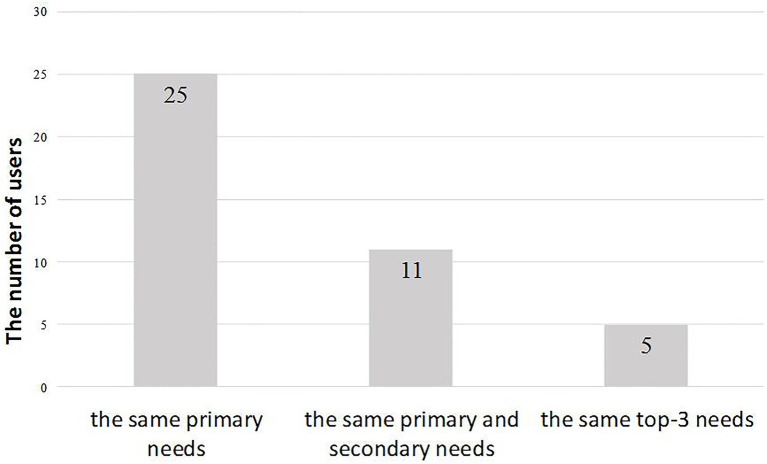
The matching degree of top-3 needs of each participant’s.

For each participant, the ranking results of questionnaire measurement and App behavior records were two different data sources to profile his/her needs. Thus, we tried to explore the consistency of needs profiles of an individual between the subjective and objective evaluations. Based on the ranking results above, we listed top-3 needs of each participant based on the questionnaire and the App usage scores, respectively, and calculated the matching degree. As shown in Figure 1, the results showed that the matching degree of primary needs reached 83.3%. For the primary and secondary needs, the matching degree fell to 36.7%, while the matching degree of the top three needs was close to the random level. These results suggested that the primary needs of the individual remained relatively highly consistent between subjective questionnaire evaluation and the scores calculated by App usage data. Also, the results suggested that for the more nonmajor needs, the matching degree of the two needs profiles was lower.

#### Difference Test of Top-3 Needs Score

**Figure 2:**
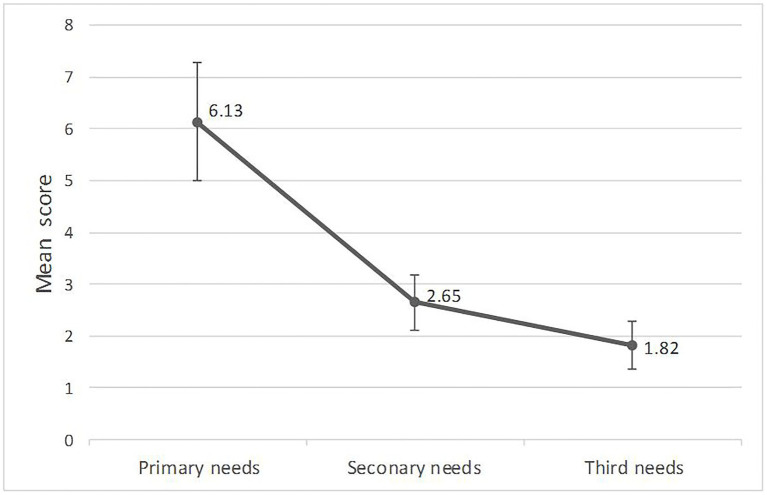
Means of top-3 needs scores of participants based on App usage data.

**Figure 3:**
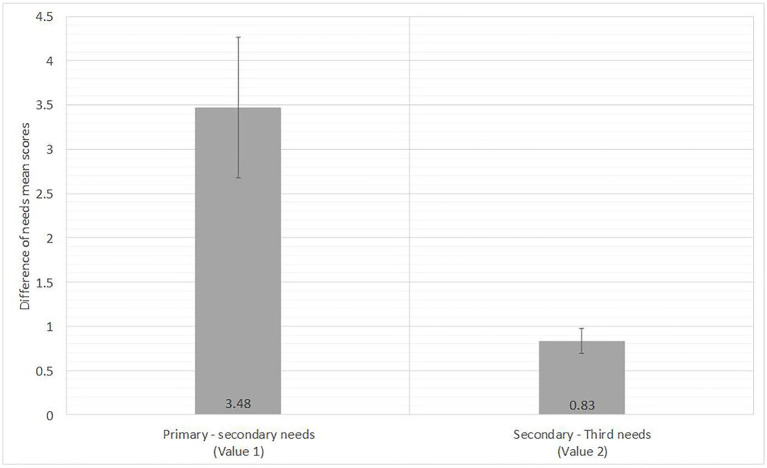
Difference test mean values of top-3 needs of participants.

In order to illustrate the rule of primary needs in the App usage behavior of an individual, we calculated mean values of top-3 needs scores of all the 30 participants, respectively, based on App usage data. Figure 2 shows the results that the mean value of the primary needs was 6.13, the mean value of the secondary needs was 2.65, and the mean value of the third needs was 1.82. In order to further explain that the proportion of the primary needs among the top-3 needs, we calculated the difference of the mean value of the primary and secondary needs (Value 1) and difference of the mean value of the secondary and third needs (Value 2). T-test was conducted and showed that Value 1 was significantly higher than Value 2, *t* (29) = 3.25, *p* < 0.01, Cohen *d* = 0.84. The results are shown in Figure 3 and suggested that the value of the primary needs had a relatively large proportion of top-3 needs of behavioral needs profile of an individual.

## Discussion

This research combined questionnaire measurement and behavior data analysis to profile the psychological needs of the App user. In study 1, based on the previous grounded work and needs theories, we constructed a questionnaire for the needs measurement of the App user and showed good reliability and validity. In study 2, based on App usage data, we calculated the needs scores of the user and compared the consistency between behavioral needs profile and questionnaire data of study 1. The results showed that the primary needs of individual user remained, approximately, highly consistent (86.7%) between subjective evaluation and the behavior data analysis.

In contrast to the general way of questionnaire construction, the theoretical framework of our study came from the previous results of GT. All items could be traced back to the interview text of the user, which enhanced the comprehensibility (Bergman and Hallberg, 2002). Although GT was classical qualitative research that did not necessarily require quantitative verification, some researchers adopted mixed method to support the model of GT with quantitative data (e.g., De Smet et al., 2019). In our research, EFA was performed and eight factors were extracted and rotated, which were consistent with the previous dimensions of the needs model of GT. A CFA further corroborated the eight-factor model compared to the unidimensional solution and verified the theoretical model of Sun et al. (2017) with questionnaire data. Although the core dimensions were consistent with the previous study of GT, the contents of some subdimensions were enriched by literature review. For example, in the previous study, the cognitive needs included “information searching” and “curiosity satisfying” categories. In this research, we enriched the “information searching” category and added measurement items based on some related studies (e.g., Gan and Li, 2018).

As for the criterion-related validity analysis results of the questionnaire in study 1, the average scores of the eight needs dimensions were positively correlated with the intention and attitudes of the usage of smartphones (*p* < 0.001). These results could be explained by UGT, that is, the higher the needs of the user was satisfied by mobile Apps usage, the stronger he/she had the intention to use the mobile phone and had the more positive usage attitudes. Specifically, in terms of the correlation coefficient, the correlation coefficients of healthy, security, and self-actualization needs were relatively lower than other needs, which was consistent with the results of GT (Sun et al., 2017). In study 2, scores of these three needs were also relatively lower than other needs in both questionnaire scores and behavior data scores.

In addition, for the cognitive needs and self-actualization needs, there were gaps between questionnaire evaluation and behavior data scores in study 2. Pun (2015) mentioned that more and more people used smartphones to obtain information for the satisfaction of their cognitive needs. In the era of fragmented reading, people could easily get a lot of information at their preferred times and places, but few of them would in fact benefit the self-growth of the individual (Liu and Huang, 2016). Therefore, the participants expected to satisfy their self-actualization needs subjectively, but most of the time they just used Apps for information collection with shallow reading. These gaps reflected the expectations of the individual that would influence his/her subjective evaluations. The results would also inspire the App developers to satisfy the self-actualization needs of the user by helping users deal with the challenges associated with fragmented reading.

In study 2, we found that the primary needs of the individual were highly consistent between questionnaire evaluation and App usage data. For the nonmajor needs, the matching degree decreased significantly. Sun et al. (2019) have found that needs-based profiles of App usage of individual were stable across different usage situations. Our research explored the stability of needs profile of the App user across self-report and usage behaviors and identified that the primary needs of the user was traits-like. These results indicated that, as the driving force, the primary needs shaped the App usage preference of the user from attitudes to behaviors. A previous study has proposed that “activated” needs of an individual shaped his/her consumption preference and purchase decision (Griskevicius and Kenrick, 2013). Gender, life experience, developmental stage, culture, and other factors were considered to affect the priority of the individual of activated needs (Kenrick et al., 2010). Our study also found that the value of primary needs of the user had a relatively larger proportion than other needs. Hirsh et al. (2012) also found similar results of personality traits of the individual. They found that, when the ads match more domain traits of the individual, they both rated it more effective and expressed greater purchase intentions. Therefore, as an application of stability, the primary needs of the user could be a core feature in the online recommendation system to profile the user and predict his/her preference.

How to profile the users precisely has been an important issue in the field of user experience (Shalala et al., 2018). Previous studies always profiled that the needs of the users completely depended on behavior record data without subjective report data of the user (e.g., Sun et al., 2019). This data-driven method can rapidly extract patterns in a short time, but it is difficult to interpret the results and to understand how they relate to theory. One way to solve the problem is to import more types of data. For example, research of Maldonado-Mahauad et al. (2018a,b) combined the behavior data collected from an online course learning platform of the users with relevant questionnaire data and identified six different online learning strategies. Our research explored to integrate the bottom-up approach of mining App behavior data with the traditional top-down approach of using validated self-reporting instruments. As a result, we designed two-dimensional needs profiles and combined two types of data sources. As an application, these needs questionnaires and behavior data analysis methods could be chosen by the user experience researchers at App development stage.

Finally, several limitations of this study need to be discussed. First, the sample size in study 2 was relatively small, because many users were cautious about the disclosure of their usage records as the data surely contained personal information and can reflect usage preference. Future studies should attempt to replicate current findings with more samples to make the results more solid. Second, for the proportions in study 2, few male participants compared to female participants authorized researchers to track their App usage behaviors. Previous studies about self-disclosure have found that females are more likely to share their information than males (e.g., Kays et al., 2012). As the proportions of the sample could not be manipulated, in future studies, we will focus on adopting a more balanced sample for more reliable results. Third, this research focused on App users in China; hence, the results may be limited to the Chinese population. Future studies could explore whether the questionnaires in study 1 and the behavior analysis procedure in study 2 have good effects in different cultures. Finally, this research only adopted two types of data sources to profile the needs of the user. More data sources, such as behavioral experiment data, comment text of the user, and so forth, could be combined to obtain a multidimensional user profile in the future.

## Data Availability Statement

The raw data supporting the conclusions of this article will be made available by the authors, without undue reservation.

## Ethics Statement

The studies involving human participants were reviewed and approved by Ethics Committee of Zhejiang University of Technology. The patients/participants provided their written informed consent to participate in this study. Written informed consent was obtained from the individual(s) for the publication of any potentially identifiable images or data included in this article.

## Author Contributions

ZS developed the study concept and drafted the manuscript. LX performed the testing and data collection. ZS and QZ performed the data analysis and interpretation. XQ provided critical revisions to the manuscript. All authors contributed to the article and approved the submitted version.

### Conflict of Interest

The authors declare that the research was conducted in the absence of any commercial or financial relationships that could be construed as a potential conflict of interest.

## Appendix

### Needs Questionnaire Items and Their Sources of Mobile App User

Please evaluate the following 28 questions according to your actual user experience of mobile Apps, from 1 (strongly disagree) to 7 (strongly agree).

Healthy Needs Satisfaction

HEN1: Some mobile Apps enable me to track my physical conditions (Taormina and Gao, 2013; Zhang et al., 2014).
HEN2: Some mobile Apps enable me to exercise the body.
HEN3: Some mobile Apps enable me to enhance physical quality.
HEN4: Some mobile Apps enable me to acquire health guidance.
HEN5: Some mobile Apps enable me to avoid physical fatigue.

Utilitarian Needs Satisfaction

UN1: Using mobile Apps enables me to handle issues easily (Liu et al., 2016).
UN2: Using mobile Apps enables me to handle issues with comprehensive functions.
UN3: Using mobile Apps enables me to find what I want effectively.
UN4: Using mobile Apps enables me to handle issues conveniently.
UN5: Using mobile Apps enables me to deal with tasks quickly (Deleted).

Security Needs Satisfaction

SEN1: Some mobile Apps enable me to feel safe and secure in some situations (Taormina and Gao, 2013).
SEN2: Some mobile Apps enable me to have a sense of trust during use.
SEN3: Some mobile Apps enable me to keep personal information securely.
SEN4: I use some mobile Apps because there will be no privacy concerns.
SEN5: I use some mobile Apps because there will be lower security-related risk (Deleted).

Low-Cost Needs Satisfaction

LCN1: Some mobile Apps enable me to find sales promotion information (Ganesh et al., 2010; Sun et al., 2017).
LCN2: Some mobile Apps enable me to get free products/services.
LCN3: Some mobile Apps enable me to find products/services at reasonable prices.
LCN4: Some mobile Apps enable me to find cheap products/services (Deleted).
LCN5: Some mobile Apps enable me to compare the prices and save money (Deleted).

Social Needs Satisfaction

SN1:Using mobile Apps enables me to belong to a group with the same interests as mine (Haridakis and Hanson, 2009).
SN2: Using mobile Apps enables me to keep in touch with friends in my real life.
SN3: Using mobile Apps enables me to express myself freely.
SN4: Using mobile Apps enables me to communicate with each other (Deleted).
SN5: Using mobile Apps enables me to maintain a daily connection with others (Deleted).

Hedonic Needs Satisfaction

HDN1: I use some mobile Apps when I have nothing better to do (Bondad-Brown et al., 2012).
HDN2: I have fun when using some mobile Apps.
HDN3: The actual process of using some mobile Apps is pleasant.
HDN4: I use some mobile Apps when I am bored.
HDN5: The actual process of using some mobile Apps is relaxed.
HDN6: Some mobile Apps enable me to get sensuous enjoyment.

Cognitive Needs Satisfaction

CN1: Some mobile Apps enable me to access the latest information (Bondad-Brown et al., 2012).
CN2: Some mobile Apps enable me to access the information I wanted to know.
CN3: I use some mobile Apps to provide information.
CN4: I use some mobile Apps to learn about how to do things I have not done before.
CN5: I use some mobile Apps to satisfy my curiosity.

Self-Actualization Needs Satisfaction

SAN1: I use some mobile Apps for career development (Kim et al., 2013).
SAN2: Some mobile Apps enable me to achieve my goals.
SAN3: Some mobile Apps enable me to get my work done.
SAN4: I use some mobile Apps to develop my capacities.
SAN5: I use some mobile Apps for financial and life instructions.
